# Application and Development of Noncontact Detection Method of α-Particles Based on Radioluminescence

**DOI:** 10.3390/s22010202

**Published:** 2021-12-28

**Authors:** Zeqian Wu, Jinxing Cheng, Mei Xu, Qingbo Wang, Ai Yu, Yue Zhang, Weiwei Wen, Youpeng Wu, Zhongfeng Tang

**Affiliations:** 1 Institute of NBC Defence, Beijing 102205, China; wuzeqian@novelmedical.cn (Z.W.); xumei@novelmedical.cn (M.X.); 2 Beijing New-High Technology Academy, Beijing 100094, China; wqb08@tsinghua.org.cn (Q.W.); yuai@novelmedical.cn (A.Y.); zhangyue@novelmedical.cn (Y.Z.); wenweiwei@novelmedical.cn (W.W.); wuyoupeng@novelmedical.cn (Y.W.); 3 Shanghai Institute of Applied Physics, Chinese Academy of Sciences, Shanghai 201800, China

**Keywords:** noncontact α-particle detection, radioluminescence, detection method, imaging method

## Abstract

The detection of α particles is of great significance in military and civil nuclear facility management. At present, the contact method is mainly used to detect α particles, but its shortcomings limit the broad application of this method. In recent years, preliminary research on noncontact α-particle detection methods has been carried out. In this paper, the theory of noncontact α-particles detection methods is introduced and studied. We also review the direct detection and imaging methods of α particles based on the different wavelengths of fluorescence photons, and analyze the application and development of this method, providing an important reference for researchers to carry out related work.

## 1. Introduction

With the development of science and technology, nuclear energy and nuclear technology are playing an increasingly important role in the social and military fields in the new era. Nuclear facilities are widely established and applied globally. Actinides are essential raw materials in nuclear weapons and nuclear reactors, and α decay is their important nuclear property.

Although α particles are weakly penetrating, they have a solid ionizing ability. If personnel accidentally inhale α radioactivity substances, they will cause severe internal exposure and significant damage to their bodies. Therefore, the monitoring of α radiation is an essential element for civil and military nuclear facilities or nuclear equipment, including radioactive waste disposal, nuclear accident emergency response, nuclear facility decommissioning, nuclear explosion monitoring, military nuclear facility management, and nuclear security. The detection of α radiation is required in all scenarios. At present, α-particle detection by sampling and in situ contact measurement is relatively mature. However, due to the short range of α particles and other characteristics and different monitoring scenarios, it is crucial to study noncontact α-particle detection technology.

Due to the short range of α particles in the air, the use of traditional α-particle detection methods requires the placement of detectors closer to the radiation source, which brings many limitations to the detection. In recent years, a noncontact detection technique based on α-particle-induced UV fluorescence effect has gradually emerged, which makes noncontact detection of α particles possible by detecting α particles ionizing air molecules to produce secondary electrons, which further excite nitrogen molecules in the air, and the nitrogen molecules are de-excited to produce fluorescence. Compared to the contact measurement techniques mainly used today, noncontact measurement has outstanding advantages in specific application scenarios. However, research into this technology is limited to laboratory studies or experimental validation, and no mature products have yet been seen.

## 2. Application of α-Particle Detection Technology

### 2.1. Application Scenarios for α-Particle Detection

#### 2.1.1. Decommissioning of Nuclear Facilities and Radioactive Waste Disposal

For radioactive waste disposal at civil and military nuclear facilities and facility decommissioning, monitoring of α radiation is an essential element. According to the Nuclear Safety Guideline [1] issued by China’s Nuclear Safety Authority, nuclear facilities include (1) nuclear power plants and installations such as nuclear power plants, nuclear thermal power plants, and nuclear steam and heat supply plants; (2) research reactors, experimental reactors, critical units, and other reactors other than nuclear power plants; (3) nuclear fuel production, processing, storage, and reprocessing (3) nuclear fuel cycle facilities such as nuclear fuel production, processing, storage, and reprocessing facilities; and (4) radioactive waste treatment, storage, and disposal facilities. The guideline requires that radioactive waste from nuclear facilities should be characterized before disposal, providing its radioactive properties, including the number and type of radionuclides, the half-life and activity concentration of each radionuclide, the total activity, the amount of heat release, and the level of α surface contamination. Moreover, it needs to be classified and preprocessed. For α-containing nuclides, the collection should be conducted separately to avoid mixing with other wastes. Therefore, the application of α detection in radioactive waste disposal is necessary.

Meanwhile, in the future, after the service life of nuclear facilities, a large number of nuclear power plants in the world will also face decommissioning, and according to the IAEA and other authorities [2,3], radiological characterization of nuclear facilities or the environment is needed before, during and after decommissioning, respectively. Monitoring and imaging of γ particles are currently possible, but γ-detection is insufficient to provide enough information on the type and source of contamination [4,5]. Actinides, as the main isotopes in the nuclear industry, release 3–9 MeV of energy when decaying, which can easily cause damage to storage materials, and after energy loss of high-energy helium nuclei, they can easily combine with electrons in surrounding materials to produce helium atoms, which can cause accumulation of helium in enclosed spaces when stored for a long time [6]. According to standards for classifying hazardous chemicals [7,8], they belong to Class II dangerous goods. Therefore, the α-decaying nuclides need to be focused on, separated, and specially preserved [3,9]. How to perform efficient detection of α-decay nuclides such as actinides and characterization of actinides at decommissioning of nuclear facilities is still a technical difficulty [4,10].

#### 2.1.2. Nuclear Power Plant Accident Response

For civil nuclear facilities such as nuclear power plants, emergency radiation monitoring also imposes requirements for detecting α radiation. While nuclear power plants are widely used, there is also the risk of accidents. The nuclear safety law requires the establishment of a nuclear accident emergency coordination committee to organize and coordinate the emergency management of nuclear accidents [11]. The concept of operational intervention level (OIL) proposed by IAEA, ICRP, and others [12,13,14] for making proper decisions in response to nuclear reactor accidents, OIL1-OIL4, requires measuring in situ α surface count rates. In the case of leaks at plutonium and uranium processing plants, the γ contamination is often not severe, and this requires measurement of α contamination in the contaminated area. Thus, α-particle detection techniques are equally worth investigating in nuclear accident emergency response.

#### 2.1.3. Management of Military Nuclear Facilities

For military nuclear facilities, such as nuclear submarines, nuclear-powered aircraft carriers, and nuclear arsenals, the need to monitor their surfaces for actinide leaks during their management relies on the detection of α radiation. Chichester et al. [15] provide methods for detecting plutonium contamination by detecting α radiation. Feener et al. [16] investigated α imaging in a nuclear warhead for verification of the prospect of its application. At the same time, for the storage and transportation of nuclear weapons, there is a possibility of accidents such as chemical explosions when the effective measurement of α contamination is needed.

#### 2.1.4. Nuclear Explosion Monitoring

In nuclear explosion monitoring, the unfissioned nuclear charge will have high α activity. If there is ^238^U material in the bomb, the share of ^239^Np and ^237^U in the fallout from the ground within ten days of the detonation is high, up to 27% or more. In their decay chains, both release α radiation. The detection of α particles is an essential element of nuclear explosion monitoring.

#### 2.1.5. Nuclear Security

The current nuclear security situation in the world is relatively severe, and nuclear smuggling is rampant. Central Asia has uncovered a world-shaking case of highly enriched uranium (HEU) smuggling in recent years. In 1995, Chechen terrorists tried to use high levels of ^137^Cs to create a terrorist incident in Moscow. Al-Qaeda terrorist groups have also acquired HEU in several ways. In the 2006 spy poisoning case, Russian agent Alexander Litvinenko was suspected of being poisoned by the administration of ^210^Po and later died from internal irradiation. Moreover, 19 days elapsed between the poisoning and the first diagnosis of ^210^Po poisoning [17]. As of December 2018, IAEA data indicate that there have been 3497 incidents such as theft or loss of nuclear or radioactive materials [18]. α-particle monitoring is an integral part of nuclear security, but the short range of α particles makes detection of α particles difficult. Kerst et al. [19] used noncontact α measurement techniques to target and study specific crime investigation scenarios.

### 2.2. Technical Means of α-Particle Detection

α-particle detection technology has developed rapidly since the 1990s, and various α detectors have gradually emerged. The application of these α detectors allows for both α-intensity measurements and α energy spectrum measurements. For α-intensity measurements, only the intensity of α particles, i.e., the number of α particles detected, can be given. In contrast, α energy spectrum measurements can provide the spectral distribution of α particles [20].

#### 2.2.1. Commonly Used α Detectors

Commonly used α detectors include gas detectors, scintillation detectors, and semiconductor detectors. Gas detectors are gas-based, and include mainly ionization chambers, positive ratio counters, and GM—counters developed more maturely. Scintillation detectors are used to convert ionization signals into fluorescence signals by the interaction of charged particles with scintillation materials, and convert fluorescence signals into electrical signals to achieve the measurement of radiation, such as CsI(Tl) and ZnS(Ag). Further, detection of alpha particles can also be performed using liquid scintillation detectors. The sample is mixed with the scintillation liquid, counted using a photomultiplier tube and the radiation signal is analyzed by waveform screening, etc. [21]. The advantage of scintillation detectors is their relatively low cost. However, semiconductor detectors have a better overall performance than scintillation detectors [22,23]. Detectors that can be applied to room temperature conditions are widely used, such as gold–silicon face-barrier detectors and passivated implanted planar silicon (PIPS). However, the detectors have the disadvantages of poor radiation resistance and have a strong temperature dependence [20]. In addition to this, solid-state nuclear track detectors (SSNTD) such as atomic nucleus emulsions have also been developed and are widely used in α-particle detection.

#### 2.2.2. α-Intensity Measurement Instrument

α-intensity meters mainly include α surface contamination meters, α lysimeter, and low background α meters. The α detection of surface contamination can be divided into two types [5]. One is a direct measurement, and the other is indirectly obtaining the information of surface contamination by measuring the wipe sample. For the measurement of α surface contamination in these two ways, international ISO standards have been developed for the treatment of various types of surface contamination [24,25,26], which describe the basic principles of evaluation methods and measurement of surface contamination, as well as the requirements for the state of the measurement instrument and data processing methods, and summarize the basic uncertainties of the two surface contamination assessment methods. The CoMo170 surface contamination checker from NUVIA, Germany, with a ZnS(Ag)-plated plastic scintillator detector, is currently used in nuclear emergency kits that have been issued to troops to enable simultaneous measurement of α and β surface contamination.

Some groups [27,28,29,30,31,32] have researched α aerosol monitors, and most of them adopt continuous sampling and real-time measurement to achieve the measurement of α intensity. At the same time, the screening of α and β can be achieved, and high sensitivity can be achieved.

Low-background α meters are mainly applied to total α measurements of samples in various scenarios. The samples are usually prepared as thin disks, or liquids are placed on metal disks [21]. Research on low-background α meters has been more extensive and well-developed.

#### 2.2.3. α Spectrometer

α energy spectrometers are commonly used to assess the content of α decay isotopes. According to the technical principle, and they can be divided into two main types: gate ionization chamber detectors and ion-injected silicon detectors, among which ion-injected silicon detectors have better resolution and are more widely used [33,34]. The current research on α spectrometers is more focused on the study of characteristic peak resolution methods [33,35,36,37,38,39,40,41,42].

### 2.3. Advantages of Noncontact α-Particle Detection

Unlike the conventional direct measurement of α particles by ionization and excitation, the noncontact α-particle detection technique is based on the secondary electrons generated by the ionization interaction of α particles with the gas in the air. The secondary electrons react further to produce fluorescent photons, and the information on α particles is obtained indirectly by the measurement of fluorescent photons.

The main advantages of using noncontact detection techniques based on the α-particle-induced UV fluorescence effect over conventional detection methods are (1) detection does not require close proximity to the α-contaminated surface, which can mitigate the hazards to personnel through better distance protection in the presence of high-radioactivity α or other types of radioactive contamination in the environment; (2) detection equipment does not require close exposure to the radiation field, which mitigates the hazards to the equipment (3) the broader range of the detection area reduces a significant amount of measurement time and cost; (4) UV light can pass through partially translucent materials (e.g., lead and glass) [4,43], allowing nondestructive detection under certain conditions where specific translucent materials are sealed, which is also suitable for analysis under conditions of high surface activity during laboratory analysis of radioactive samples and for packaging in UV-transparent boxes for the examination of unknown or potentially hazardous samples [4]. It can be seen that the method of noncontact detection of α particles has significant advantages, and the study of noncontact detection methods is of great importance.

The phenomenon that α particles induce air luminescence was first proposed by Huggins et al. in 1903 [44], demonstrating that air fluorescence comes from the de-excitation of the 2P and 1N extranuclear electron layers’ nitrogen [45]. In recent years, several aspects of noncontact detection techniques based on the α-particle-induced UV fluorescence effect have been investigated, focusing on direct detection and imaging. The airborne UV fluorescence information utilized comes from the UVA/UVB band and the UVC band, respectively. The strengths and weaknesses of these methods are shown in Table 1.

## 3. Principle of Noncontact α-Particle Detection Technology

The α-particle-induced air fluorescence effect is mainly caused by the interaction of secondary electrons with air molecules. The main components of air are nitrogen and oxygen, and fluorescent photons are mainly generated by collisions with nitrogen molecules [46], with the share of luminescence from 300 nm to 400 nm wavelengths of UV light accounting for 95% of the total radioluminescence [47,48]. In 2016, Thompson et al. [49] described the physical process of α-particle-induced fluorescence effect as an air fluorescence model AFM (Air Fluorescence Model), and the simulation calculation results of this model are very close to the experimental results of Sand et al., 2014 [50], and the theoretical model can be considered as reasonable. This Air Fluorescence Model consists of three main stages: the first stage consists of the interaction between α particles and air molecules to produce secondary electrons; in the second stage, the secondary electrons continue to excite atmospheric molecules; in the last stage, the nitrogen molecules and ions undergo 2P and 1N de-excitation, releasing UV light mainly through $C^{3}\Pi_{u}\to B^{3}\Pi_{g}$ and $B^{2}\sum_{u}^{+}\to X^{2}\sum_{g}^{+}$. For oxygen, the interaction with secondary electrons produces excited oxygen atoms, which then spontaneously degenerate and are the main component of the oxygen emission spectrum. These three phases will be analyzed in turn below.

### 3.1. Secondary Electron Generation Phase by Ionization of α-Particles

The first stage is the one in which α particles interact with air molecules and produce secondary electrons. The α particles released by decay interact with the components of the air as they travel through it. The charged particle–matter interaction consists of several primary forms: inelastic collisions, radiative interactions, elastic scattering, nuclear interactions, and electron pair production. Through the various interactions, there will be a gradual loss of energy. α-particles collide inelastically with electrons outside the nucleus of a dielectric atom, and the loss of energy after a unit path is called the stopping power. Bethe et al. [51] gave an expression for the mass stopping power, Bethe’s formula:

(1) $$-\frac{dE}{dx}=\frac{4\pi z^{2}e^{4}ZN}{m_{0}v^{2}}[\ln(\frac{2m_{0}v^{2}}{I})-\ln(1-\beta^{2})-\beta^{2}]$$

where *v* is the particle flight velocity, *Z* is the atomic number of the medium, *ze* is the charge of the charged particle, *m*_0_ is the rest mass of the electron, *β* is *v/c*, *c* is the speed of light, *N* is the number of atoms per unit volume in the medium, and *I* is the average ionization potential, which represents the average of the excitation and ionization energies of the electrons in each shell layer.

Since the maximum possible speed of travel of α particles is 1.7 × 10^7^m/s, which is about 6% of the speed of light, relativistic effects can be neglected when studying α particles [12], and Bethe’s formula can be simplified to, for example,

(2) $$-\frac{dE}{dx}=\frac{4\pi z^{2}e^{4}ZN}{m_{0}v^{2}}[\ln(\frac{2m_{0}v^{2}}{I})]$$

The formula is only applicable to the high-energy region, for the energy region near 500*I*, the ionization-loss process is very complex and affected by a variety of factors. There is no better theory to describe it which can be understood qualitatively; the reduction in the flight speed is conducive to the transfer of energy, but the speed is below a specific value, the inner shell electrons of atoms in the air no longer participate in ionization collisions, and the probability of capturing electrons increases, and the air atomic effective charge decreases, so the ionization collision energy loss decreases [52].

For energy regions below this region, Lindhard et al. [53] proposed the LSS theory, giving the formula for the stopping power as:

(3) $$-\frac{dE}{dx}=z^{\frac{1}{6}}8\pi e^{2}Na_{0}\frac{zZ}{{\left(z^{\frac{2}{3}}+Z^{\frac{2}{3}}\right)}^{\frac{3}{2}}}\cdot \frac{v}{v_{0}}$$

where *a*_0_ is the Bohr radius. For lower energy regions, elastic collisions become the dominant energy-loss process and can be described using Rutherford scattering theory [52].

Various simulation tools have been developed to model the interaction processes of α particles with matter. SRIM [54] is a program based on theoretical models and experimental data to simulate the energy deposition distribution of energetic particles in a medium, allowing the user to perform flexible modeling calculations. The International Commission also gives relevant results on Radiation Units and Measurements (ICRU) [55,56] and the National Institute of Standards and Technology (NIST) [57], among others, and the differences are small.

When all the kinetic energy of the α particle is lost, it will stop in the air, at which point the distance the α particle travels in the air is defined as the range, and the range expression is:

(4) $$R=\int_{E_{0}}^{0}dE/(-dE/dx)$$

where *−dE*/*dx* is the stopping power. Due to the relative complexity of the theoretical part of the calculation, an empirical formula for the variation of range with energy was obtained from the experiment:

(5) $$R=(0.005E+0.285)E^{\frac{3}{2}}$$

where the range *R* is in cm, and the energy *E* is in MeV. This empirical formula gives a range of about 36 mm for α particles with an energy of 5.1 MeV in standard air, which is closer to the range result of 38 mm obtained by other researchers from ^239^Pu experiments at temperatures between 22 °C and 28 °C [58]. The error may be caused by the difference in temperature. For 6.1 MeV α particles, the range calculated using the empirical formula is about 48 mm, which better agrees with the other simulated experimental result of 50 mm [59].

**Figure 1:**
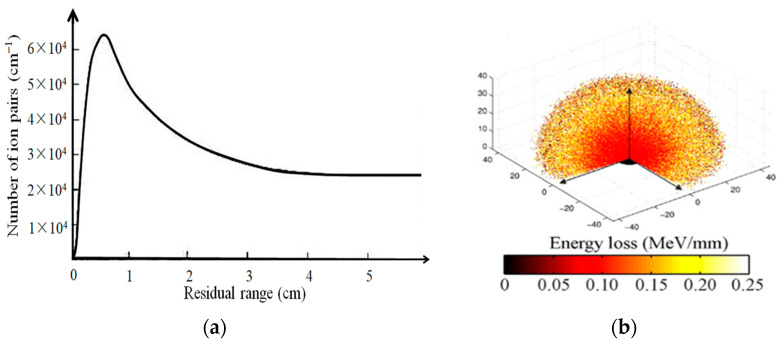
Comparison of Bragg curves of α particles emitted by ^210^Po and simulation of the 3D trajectory of α particles emitted by ^239^Pu. (**a**) Bragg curves for α particles emitted by ^210^Po (**b**) Simulation of 3D trajectories for α particles emitted by ^239^Pu [58]. Figure 1b reprinted with the permission of Elsevier.

As a charged particle travels through air, it continuously collides inelastically with electrons outside the nucleus of an air atom, exciting and ionizing it. Specific ionization is the number of electron–ion pairs produced per unit distance traveled. The Bragg curve describes the variation of specific ionization with the remaining range, and the Bragg curve for α particles emitted by ^210^Po is shown in Figure 1a [52].

It can be seen that as the remaining range decreases, the specific ionization increases first and begins to decrease rapidly after reaching a peak. This can be explained by the gradual neutralization of alpha particle by its continuous capture of electrons. [52]. It was shown that for both α particles and electrons, the average energy to create a charged particle produce an electron–ion pair in the air is independent of the type and energy of the charged particle. Ideally, the emission range of an α point source is a sphere [60,61]. Sand et al. [58] simulated energy-loss diagrams for the α-particle trajectories produced by ^239^Pu decay, and the results coincided with the Bragg curve profile. The inner ring is darker due to the higher number of α particles per unit volume in the inner ring.

In their simulations, Thompson et al. [49] used Rudd’s semiempirical model [62] to describe the ionization process of α particles in air, and implemented it by Geant4 to produce secondary electron spectra.

### 3.2. Secondary Electron Ionization Air Phase

The second stage is the excitation of air by secondary electrons. In the field of cosmic rays, extensive research has been carried out in astrophysics to produce fluorescence in the air using the ionization effect of electrons [63,64,65]. The types of electron–matter interactions can be mainly classified as ionizing and radiative energy losses. When ionizing radiation loss occurs, the mechanism of action is similar to that of an α-particle–matter interaction. For high-energy electrons, Arqueros et al. [63] developed the Opal formula [66] to provide a semiempirical formula for the ionization cross-section. For lower-energy electrons, the inelastic collision cross-section needs to be considered, and Itikawa et al. took the average value from different experiments as the ionization cross-section [67]. Air is excited by secondary electrons at this stage, with about 78% of nitrogen and 21% of oxygen in the air. Therefore, the mechanism and results of the interaction of molecules with secondary electrons occurring with nitrogen and oxygen will be investigated below.

#### 3.2.1. Interaction of Secondary Electrons with Nitrogen

According to the electronic energy band system of the nitrogen molecule, nitrogen lies in the electron interactions that result in a variety of secondary particles, including the excited state of the nitrogen molecule N_2_, the nitrogen molecule ion N_2_^+^, the nitrogen atom N, and the nitric oxide NO [68,69]. The emission spectra produced by N_2_ molecules include the N_2_ first positive systems (FPS), second positive systems (SPS), Herman’s IR band system, and Gaydon’s Green band system. In contrast, the emission spectra produced by N_2_^+^ in the secondary particles consist mainly of the first negative and second positive band systems.

Since the fluorescence in the 300–400 nm range produced by nitrogen is mainly from the second positive system of N_2_ and the first negative system of N_2_^+^ emission spectra [48,70,71], the second positive system of N_2_ and the first negative system of N_2_^+^ were used as the main objects of study.

(1) $N_{2}(C^{3}\Pi_{u})$ State

$N_{2}(C^{3}\Pi_{u})$ are generated from three main parts. The first part is generated by electron-by-electron excitation [72], and the second type is $N_{2}(C^{3}\Pi_{u})$, achieved by generating intermediate substable states $N_{2}(A^{3}\sum_{u}^{+})$, which continue to be generated [68]. The main reactions are that of

(6) $$N_{2}(X^{1}\Sigma_{g}^{+})+e=N_{2}(A^{3}\Sigma_{u}^{+})+e^{-}$$

(7) $$N_{2}(A^{1}\Sigma_{u}^{+})+e=N_{2}(C^{3}\Pi_{u})+e^{-}$$

The last part comes from the direct excitation of high-energy electrons such that

(8) $$N_{2}(X^{1}\Sigma_{g}^{+})+e(fast)=N_{2}(C^{3}\Pi_{u})+e^{-}$$

(2) $N_{2}{}^{+}(B^{2}\sum_{u}^{+})$ State

$N_{2}{}^{+}(B^{2}\sum_{u}^{+})$ The state is derived from nitrogen being excited directly from the ground state, or first into the intermediate state $N_{2}{}^{+}(X^{2}\sum_{g}^{+})$, the

(9) $$N_{2}(X^{1}\sum_{g}^{+})+e^{-}\to N_{2}{}^{+}(X^{2}\sum_{g}^{+})+e^{-}$$

(10) $$N_{2}(A^{3}\sum_{u}^{+})+e^{-}\to N_{2}{}^{+}(X^{2}\sum_{g}^{+})+e^{-}$$

(11) $$N_{2}(A^{3}\sum_{u}^{+})+N_{2}(A^{3}\sum_{u}^{+})\to N_{2}(X^{1}\sum_{g}^{+})+N_{2}{}^{+}(X^{2}\sum_{g}^{+})+e^{-}$$

(12) $$N_{2}({a^{\prime }}^{1}\sum_{u}^{+})+N_{2}({a^{\prime }}^{1}\sum_{u}^{+})\to N_{2}(X^{1}\sum_{g}^{+})+N_{2}{}^{+}(X^{2}\sum_{g}^{+})+e^{-}$$

(13) $$N_{2}(A^{3}\sum_{u}^{+})+N_{2}({a^{\prime }}^{1}\sum_{u}^{+})\to N_{2}(X^{1}\sum_{g}^{+})+N_{2}{}^{+}(X^{2}\sum_{g}^{+})+e^{-}$$

is further excited to $N_{2}{}^{+}(B^{2}\sum_{u}^{+})$ [68]

(14) $$N_{2}{}^{+}(X^{2}\sum_{g}^{+})+e^{-}\to N_{2}{}^{+}(B^{2}\sum_{u}^{+})+e^{-}$$

(15) $$N_{2}{}^{+}(X^{2}\sum_{g}^{+})+N_{2}{}^{+}(X^{1}\sum_{g}^{+})\to N_{2}{}^{+}(X^{1}\sum_{g}^{+})+N_{2}{}^{+}(B^{2}\sum_{u}^{+})$$

#### 3.2.2. Interaction of Secondary Electrons with Oxygen

Oxygen lies in the electron interactions that result in the formation of various secondary particles such as excited-state oxygen molecules O_2_, oxygen molecule ions O_2_^+,^ and excited-state oxygen atoms O. In the emission spectrum of oxygen, the spectral emission lines of oxygen atoms are the main components of the emission spectrum of oxygen. Oxygen molecules in different states will interact with electrons to produce oxygen atoms. The states produce oxygen atoms $O_{2}(X^{3}\Sigma_{g}^{-})$ through the following reactions [73]

(16) O2(X3Σg−)+e−→O(P3)+O(P3)+e−

(17) O2(X3Σg−)+e−→O(P3)+O(D1)+e−

(18) O2(X3Σg−)+e−→O(D1)+O(D1)+e−

The $O_{2}(a^{1}△)$ state produces oxygen atoms through the reaction of

(19) O2(a1△)+e−→O(P3)+O(P3)+e−

The $O_{2}(A^{3}\sum_{u}^{+})$ state produces oxygen atoms through the reaction of

(20) O2(A3∑u+)+e−→O(P3)+O(P3)+e−

(21) O2(A3∑u+)+e−→O(P3)+O(D1)+e−

The $O_{2}(b^{1}\sum_{g}^{+})$ state produces oxygen atoms through the reaction of

(22) O2(b1∑g+)+e−→O(P3)+O(P3)+e−

(23) O2(b1∑g+)+e−→O(P3)+O(D1)+e−

The oxygen atom in the generated ground state is further excited by interactions with electrons, by

(24) O(P3)+e−→O(D1)+e−

(25) O(D1)+e−→O(S1)+e−

the excited oxygen atom can further collide with the substable oxygen molecule to produce oxygen atoms in the higher energy state

(26) O(S1)+O2(B3∑u−)→O(S3o)+O2(X3∑g−)

(27) O(S1)+O2(A3∑u−)→O(S3o)+O2(X3∑g−)

(28) O(S3o)+O(D1)→O(P3)+O(S3o)

### 3.3. Air-Generated Fluorescence Phase

#### 3.3.1. Fluorescence Fundamentals

The final stage is the fluorescence process of air. The emission and absorption spectra of air molecules are determined by their electronic structure [59,74]. The process is governed by the principles of diatomic molecular spectroscopy [9]. When air interacts with secondary electrons, it absorbs energy and enters the excited state, which has a different spin multiplet than the ground state. The light produced by the radiative leap between states with the same multiplicity is called fluorescence. The light emitted when the triplet excited state leaps to a single ground state are phosphorescence. Since the leap of the phosphorescent triplet state to the single heavy state is a forbidden ring leap, the spin wave functions corresponding to the initial and final states are orthogonal to each other, and the leap integral is zero [75] reflected by a fragile intensity and slow emission rate.

Molecular spectra are influenced by a combination of their relatively complex spatial structure and forms of motion. Covalent or ionic bonds link atoms in a molecule. There are different forms of motion within it, such as the motion of electrons relative to the nucleus, the vibration of the nucleus, and the rotation around its center of gravity, which cause the molecule to have three quantized energies, the electron energy *E_e_*, the vibration energy *E_v_*, and the rotation energy *E_r_*:

(29) $$E=E_{e}+E_{v}+E_{r}$$

Only when the rotational energy level jumps does it correspond to the rotational spectrum, which corresponds to the microwave and far-infrared regions of the spectrum; when the vibrational energy level jumps, it is accompanied by a jump in the rotational energy level, called the vibrational spectrum, which corresponds to the infrared region; when the electron jumps between two energy levels, it corresponds to the UV region of the spectrum.

The energy scales for the three different types of energy leaps are [48,68]:

(30) $$E_{e}\gg E_{v}\gg E_{r}$$

The difference in mass between the electron and the nucleus is significant. According to the Born–Oppenheimer approximation, the nucleus is not disturbed when the electron undergoes rapid changes in its orbit, and the process by which the electron adjusts its state of motion can be ignored when the distribution of the nucleus undergoes small changes, i.e., the motion of the nucleus is adiabatic concerning the electron energy and can be treated independently of each other. Therefore, the molecular state can be expressed as [48]

(31) $$\Psi =\psi_{e}\cdot \psi_{v}\cdot \psi_{r}$$

The relative magnitudes of these three energies can be found by the semiempirical formula [48]

(32) $$E_{e}:E_{v}:E_{r}=1:\sqrt{\frac{m}{M}}:\frac{m}{M}$$

where *m* is the mass of the electron and *M* is the approximate mass of the nucleus.

In order to obtain the position of the peaks in the emission spectrum, an analysis of the photon energy is required. Based on the conditions of the Born–Oppenheimer approximation, for diatomic molecules, the wave number of the lepton-generating photons can be expressed as [68]

(33) $$\nu =T^{\prime }-T^{\prime \prime }=({T_{e}}^{\prime }-{T_{e}}^{\prime \prime })+({T_{v}}^{\prime }-{T_{v}}^{\prime \prime })+({T_{r}}^{\prime }-{T_{r}}^{\prime \prime })=\nu_{e}+\nu_{v}+\nu_{r}$$

where *ν_e_* is the wave number of photons produced by the electron energy level leap, *ν_ν_* is the wave number of photons produced by the vibrational energy level leap, and *ν_r_* is the wave number of photons produced by the rotational energy level leap. The intensity of the atomic emission lines can be obtained by

(34) $$I_{emission}^{nm}=N_{n}hcv_{nm}A_{nm}=\frac{64N_{n}c\pi^{4}}{3}v_{nm}^{4}{\left|R^{nm}\right|}^{2}$$

where *ν_nm_* is the wavenumber of the released photon that jumps from the *n* state to the *m* state, *c* is the speed of light, *h* is Planck’s constant, *A_nm_* is the Einstein factor for the jump from the *n* state to the *m* state, and *N_n_* is the number of atoms.

According to the Franck–Condon Principle, the time scale of the leap between electronic states in a molecule is very short compared to the faster change in nuclear spacing. Thus, the nuclear spacing or momentum can be considered constant when a leap occurs, and shows an abrupt change on the potential energy profile graph. The total lepton moment can be expressed as

(35) $$\langle \Psi^{\prime \prime }(r,R)|M(r)|\Psi^{\prime }(r,R)\rangle \approx \langle \psi^{\prime \prime }(r,R_{v^{\prime },v^{\prime \prime }})|M(r)|\psi^{\prime }(r,R_{v^{\prime },v^{\prime \prime }})\rangle \cdot \langle \chi^{\prime \prime }(R)|\chi^{\prime }(R)\rangle$$

where $\langle \Psi^{\prime \prime }(r,R)|$ is the final state of the molecular leap, $|\Psi^{\prime }(r,R)\rangle$ is the starting state of the leap, ψ(r,R) is the wave function of the electron, χ(R) is the wave function of the nucleus, and $R_{v^{\prime },v^{\prime \prime }}$ represents the nuclear spacing. The first term on the right-hand side of the equation is expressed as the moment of the electron leap at a nuclear spacing of $R_{v^{\prime },v^{\prime \prime }}$, and the second term represents the overlap integral between the initial and final states of the leap [48], the square of which is known as the Franck–Condon factor

(36) $$q_{v^{\prime \prime }v^{\prime \prime }}={\left|\langle \chi^{\prime \prime }(R)|\chi^{\prime }(R)\rangle \right|}^{2}$$

When air is excited by secondary electrons [9,76], its de-excitation process releases photons of longer wavelengths. From the point of view of atomic molecular spectroscopy, according to the Franck–Condon principle, the excitation of air to higher vibrational states by secondary electrons, after a series of radiation-free de-excitation processes, leads to an increase in the nuclear spacing, triggering a change in the electronic state, which in turn releases a small amount of energy by emitting longer wavelength photons.

The leap of a microscopic particle from a higher to a lower energy level consists of two main types of spontaneous and exciting radiation. The spontaneous radiation of a molecule in an excited state is often described using the Einstein coefficient, which is proportional to the square of the leap moment. Using Equations (1)–(35) to represent the leap moment, one obtains that

(37) $$A_{v^{\prime },v^{\prime \prime }}\propto q_{v^{\prime },v^{\prime \prime }}\cdot {\left|\langle \psi^{\prime \prime }(r,R_{v^{\prime },v^{\prime \prime }})|M(r)|\psi^{\prime }(r,R_{v^{\prime },v^{\prime \prime }})\rangle \right|}^{2}$$

The Einstein coefficient represents the probability of a molecule making a spontaneous jump:

(38) $$\frac{dN(t)}{dt}=-A_{v^{\prime },v^{\prime \prime }}N(t)$$

where *N*(*t*) denotes the number of molecules in the excited state at time *t* and *A_v_*_′,*v*__″_ denotes the Einstein coefficients for the spontaneous jump from state *ν*′ to state *ν*″. Solving this differential equation yields

(39) $$N(t)=N(0)e^{-A_{v^{\prime },v^{\prime \prime }}t}$$

where *N*(0) represents the number of molecules in the excited state at the initial time. The value of this factor is independent of the radiation field. It is only inversely proportional to the average lifetime of the molecule at the initial energy level, which can be obtained by looking up the table.

#### 3.3.2. Fluorescence Processes of Nitrogen and Oxygen

**Figure 2:**
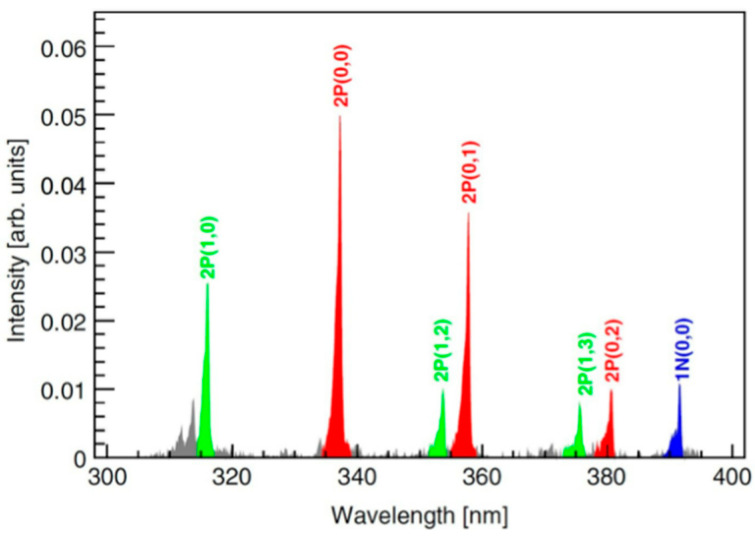
Air fluorescence spectrum in the 300–400 nm range. Peaks of different energies represent radioluminescence photons produced by different leapfrogging modes [48]. This figure is reprinted with the permission of Elsevier.

The fluorescence of air in the 300–400 nm range comes mainly from the emission spectra of the 1N and 2P systems of nitrogen [48,70], which is the most significant component of air, shown in red, blue, and green in Figure 2, while the other components are shown in grey [48]. The fluorescence process can be expressed as

(40) $$2P(v^{\prime },v^{\prime \prime }):C^{3}\Pi_{u}(v^{\prime })\to B^{3}\Pi_{g}(v^{\prime \prime })$$

(41) $$1N(v^{\prime },v^{\prime }):B^{2}\Sigma_{u}^{+}(v^{\prime })\to X^{2}\Sigma_{g}^{+}(v^{\prime \prime })$$

The generated $N_{2}(C^{3}\Pi_{u})$ generates the emission spectrum of the second positive band system of N_2_ by the following leap

(42) $$N_{2}(C^{3}\Pi_{u})\to N_{2}(B^{3}\Pi_{g})+\gamma$$

The generated $N_{2}{}^{+}(B^{2}\sum_{u}^{+})$ generates the emission spectrum of the first negative band system of $N_{2}{}^{+}$ by the following leap

(43) $$N_{2}{}^{+}(B^{2}\sum_{u}^{+})\to N_{2}{}^{+}(X^{2}\sum_{g}^{+})+\gamma$$

The fluorescence process of oxygen was verified in an oxygen emission spectroscopy experiment at a concentration of 99.995%, showing that the most intense part of the oxygen fluorescence spectrum is the 777.4 nm line (S5o,9.15eV)→(P5,10.74eV) generated by and the 844.7 nm line generated by (S3o,9.52eV)→(P3,10.99eV) [68].

Brett et al. [77] investigated the effect of α-particle-induced fluorescence from Ar, N_2_, O_2_, and dry air at standard atmospheric pressure with a single detector in the wavelength range of 250–1100 nm, and corrected for the energy loss from the generated fluorescence photons passing through the detector, and found that in dry air, the N_2_ contribution to the luminescence brightness contribution is much more significant than that of O_2_.

#### 3.3.3. Quantitative Analysis of Luminous Output

The degradation of molecules from the excited state takes place not only in the form of the release of photons but also through the nonradiative release of energy to complete the degradation. There is also an energy transfer from mutual collisions between molecules in the air, known as bursts. The presence of oxygen in air produces a burst in the fluorescence of nitrogen, reducing its luminescence efficiency [4,60,78].

In order to calculate the luminescence yield of the α-particle-induced fluorescence effect in air, different teams have investigated the problem from both experimental and theoretical perspectives.

Sand et al. [50] built a conformal detection platform based on detecting ^239^Pu radioactive sources. The photomultiplier will detect the probability P that an α-particle-induced generated photon can be expressed as

(44) P=Y⋅QE⋅Ω

where *Y* is the luminescence yield, *QE* is the quantum efficiency of the photomultiplier tube, and Ω is the geometric efficiency of the device, which can be obtained by calculating the ratio of the surface areas of equivalent spheres.

Then, based on the α surface count rate *N_S+B_* detected by the device, we conduct a least-squares calculation using the following equation

(45) $$N_{S+B}=A_{S}\cdot P+N_{B}$$

where *A_s_* is the count rate of α-particle emission from the surface of the ^239^Pu source and *N_B_* is the background count rate. *A_s_* is a known quantity and *N_S+B_* can be obtained directly by experiments. Experiments are conducted separately at different distances, and a series of data is obtained. A least-squares fit to these data gives an estimate of *P*, which in turn leads to a calculation of the luminescence yield for the α-particle-induced fluorescence effect on the air.

Sand et al. [50] experimentally found that α particles produce 19.4 photons for every 1 MeV of energy released by α particles in the air at atmospheric pressure, 43% relative humidity and 22.0 °C, and that the number of photons produced is linearly proportional to the energy released in the energy range from 0.3 MeV to 5.1 MeV.

Thompson et al. [49] calculated using the theoretical air fluorescence model AFM that 18.9 photons are produced for every 1 MeV of energy released by α particle in the air under this environmental condition, which is very close to the energy range of 0.3 MeV to 5.1 MeV calculated by Sand et al., and an exponential relationship describing the α-particle energy versus the number of photons produced would be more accurate than a linear relationship when extending the air fluorescence model to the energy range of 0.1 MeV to 10.0 MeV.

Meanwhile, Thompson et al. found that air has a strong stopping power for α particles at the same energy compared to β particles and γ particles, which is about 1000 times higher than that of β particles [9,56]. Based on this property, Thompson et al. performed Monte Carlo simulations of the induced fluorescence effects of α particles, β particles, and γ particles, respectively. They concluded that α particles are more easily and precisely localized.

## 4. Development of Noncontact α-Particle Detection Technology

Since the share of luminescence at 300–400 nm wavelengths in α-induced radioluminescence accounts for 95% of the total radioluminescence, the primary research is currently focused on the acquisition, processing, and utilization of UV information in this band. Of these, 315–400 nm is the UVA band, and 280–315 nm is the UVB band.

Most of the α radioluminescence is in the wavelength range of 300–400 nm, while the solar irradiation in this band can reach (2–8) × 10^–2^ W·cm^2^·nm^–1^, and even the UV luminescence in this band in the nighttime environment is much larger than the luminescence from public α-radiation sources [47,78]. In the natural environment, the background luminescence is much larger than the characteristic luminescence, and the characteristic information is masked by noise, making it difficult to use the luminescence in the UVA/UVB band for α-radiation detection, so researchers have also carried out UV detection based on the UVC band. The UVC band refers to UV radiation with wavelengths between 200–280 nm, and light from the Sun in this band is absorbed by the atmosphere and the ozone layer. Thus, the background signal in this band on Earth is small [79]. There is no interference with the characteristic signal. Hence, UV-detection devices based on this band are often also called day-blind detectors. However, since only a tiny fraction of the luminous spectrum of air lies in the day-blind region, the detection limit of the instrument would increase by two orders of magnitude if only the UV photons in the UVC band were used as the characteristic signal for noncontact α detection in natural light [19].

### 4.1. α-Particle Detection Based on UVA/UVB Bands

In 2010, Leybourne et al. [80] achieved the detection of a ^210^Po source of 37 MBq at a distance of 150 m from the detector at night, within a data acquisition time of less than 1 min. However, this experiment could only demonstrate the presence of α sources at a large resolution, with poor localization accuracy and without quantitative analysis.

Inrig et al. [59] achieved detection of an α source of 37 kBq using a UV-band-free source for illumination at 1.5 m from the detector for 10 s. This measurement is more accurate but requires a more demanding environment, and it is difficult to perform outdoor measurements. In 2016, Sand et al. [81] conducted separate experiments using superb basic and cesium telluride photocathodes in the presence or absence of UV-band illumination. They showed that the minimum detectable activity for α sources without UV illumination at a distance of 1 m and a measurement time of 10 s was 4 kBq. The minimum detectable activity for α sources under normal illumination conditions is 800 kBq.

**Figure 3:**
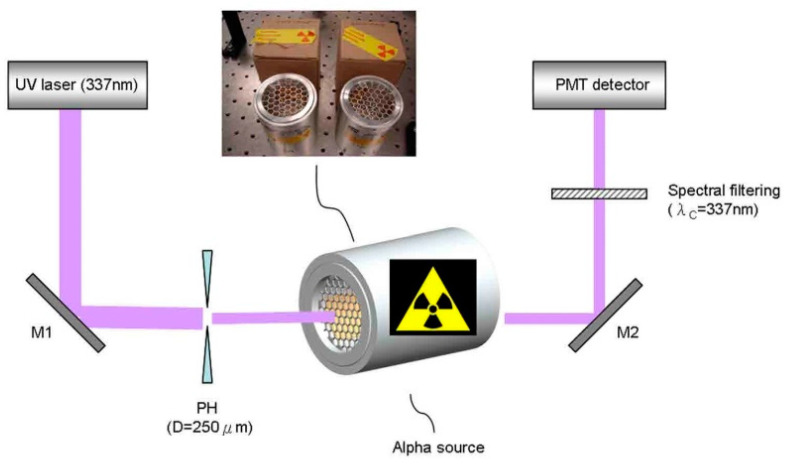
In this detection system, the α particles released by the radioactive source are optically focused, filtered, photomultiplied, and electronically processed, and the information is collected by a computer system [79]. This figure is reprinted with the permission of AIP Publishing.

The coexistence of multiple radioactive sources is inevitable when radiometric detection of α is carried out in practice. To investigate the effect of the presence of β and γ radiation on the detection of α, Baschenko et al. [47] compared the brightness of three types of ray-induced radioluminescence by comparing the intensity ratios between α-, β- and γ-induced radioluminescence of 1:10^−8^:10^−10^. It can be shown that the interference with the detection of α radioactivity is less in the case of less high levels of β and γ radiation. An example of a detection system for noncontact α detection is shown in Figure 3.

Since these systems need to be under conditions of no background light, low background light, or special light, the difficulty of controlling the light conditions currently limits the use of these methods in this field. Therefore, the technique is being studied and improved, mainly through two efforts, to enhance fluorescence efficiency and filtering to reduce background interference.

For improving the fluorescence efficiency, the presence of oxygen in the air causes a burst in the luminescence of nitrogen, which can be enhanced by removing oxygen. In 2010, Hannuksela et al. [60,74] studied luminescence of the environment after the removal of oxygen and detected ^241^Am of 10 kBq in the presence of nitrogen only, and the count rate of the detector was increased from 150 cps to 650 cps. In 2012, in a study by Ihantola et al. [82], for an ^241^Am source of 4.2 kBq at a distance of 15.7 cm, the UV signal intensity in the nitrogen-only environment was about five times higher than the signal intensity in the air.

For filtering methods, the most commonly used method is the direct use of a filter. This filter allows only a specific wavelength of light to pass through. By selecting that specific wavelength in the UV region of our interest, most of the interference from other bands of particles can be blocked out, thus reducing the background. In 2010, Sand et al. [60] used a 300–340 nm filter, containing the most substantial peak at 337 nm. In 2005, Lamadie et al. [4] used a bandpass filter of 280–400 nm, and Ivanov et al. [83] chose a filter with a selected pass wavelength range of 280–390 nm.

In addition to direct filtering of particles at specific wavelengths using filters, Sand et al. [60] also used a conformal measurement method for filtering. By combining several filters and then spectrophotographing the primary filtered rays, one of the beams is selected to filter out the nonconcern band 298–303 nm alone. The fluorescence in this band is extracted separately as noise. The spectrum can be further cleaned up and the signal-to-noise ratio improved by subtracting this noise using another beam of rays after spectroscopy. After filtering using this method, a 1.2 kBq point source at a distance of 40 cm from the detector can be identified within 1 s. In addition, an alternative way of conformal measurement was also carried out to achieve better filtering by increasing the number of photomultiplier tubes for conformal measurement.

### 4.2. α-Particle Detection Based on UVC Band

In 2013, Roberts et al. [84] investigated α-particle detection based on α-induced radioluminescence in the UVC band, using a photomultiplier tube and bandpass filter for a ^210^Po source, demonstrating the presence of UVC photons with the ability to detect these photons, but not quantifying the photon yields.

In 2017, Crompton et al. [85] detected photons in the 180–260 nm wavelength range induced by a ^210^Po source with an intensity of 6.95 MBq at a distance of 20 mm from the detector, based on a flame detector (UVTRON) model R9533 manufactured by Hamamatsu, Japan, which was investigated to achieve daylight detection of α particles in the UVC-band detection in the UVC band. Even though the experiments were carried out relatively close to the detector, it was tentatively demonstrated that a more noncontact α detection was possible after some electronics processing. Later experiments showed that the detector is susceptible to interference from susceptible β and γ radiation, limiting its use in scenarios where multiple types of contaminant sources are present simultaneously [86]. In 2018, Crompton et al. [87] placed gas pipes near a 6.95 MBq ^210^Po radioactive source, and when blown with Ar, Xe, Ne, _N2_, and Kr gases, respectively, observed detected signal changes in the UVC band and concluded that all signals increased when the above gases were blown in, and the most significant enhancement effect on the signal was observed when Xe was blown in. Sand et al. [81] experimentally obtained that two orders of magnitude of signal enhancement could be achieved in the deep-UV region (below 260 nm) if nitrogen and argon were used for blowing.

Due to the low number of photons in the UVC band produced by α induction, in 2017, Kerst et al. [88] noted that in the UVC band, only nitrogen in the air could produce UV light that can be detected. Researchers have carried out studies on how to increase the number of UVC photons. Since the emission spectrum of NO lies in the day-blind region, Kerst et al. [89] investigated the effect of the concentration of nitric oxide in nitrogen on the luminescence yield. They had the best luminescence enhancement when the concentration of nitric oxide in nitrogen was 50 ppm. The intensity of the most intense spectral line in nitric oxide was 25 times higher than in nitrogen.

In 2019, Kerst et al. [90] investigated the enhancement of nitric oxide luminescence using nitrogen purging. The radioluminescence of nitric oxide is mainly in the wavelength range of 200–300 nm. The low background in this band on Earth makes noncontact detection using UVC photons possible under daylight conditions. However, due to the burst effect of oxygen and water vapor, the already small number of UVC photons is further reduced, making it more challenging to effectively capture UVC photons for radioluminescence detection. The radioluminescence of carbon monoxide was enhanced using nitrogen purging, and the radioluminescence mechanism and aerodynamic processes were investigated, leading to the conclusion that the method can be used in nuclear industry gloveboxes containing N_2_, and that NO concentrations of 100–1 ppm can be used for remote detection of α particles under standard illumination conditions. The above methods for increasing UVC photons have been investigated from the perspective of luminescence mechanisms and electronics to improve the efficiency of detecting UVC photons. Shaw et al. [91] compared five different available detection techniques. They concluded that the Geiger-mode avalanche photodiode detector (GM-APD) was superior to the use of CCD cameras or PMTs for UVC photon detection, for UV quantum efficiency at wavelengths of 270 nm is better.

## 5. Development of α-Particle Imaging Technology

### 5.1. α Imaging Based on UVA/UVB Bands

α imaging based on the UVA/UVB band was first demonstrated with the film [44]. Because of the substantial UV interference from the background in the UVA/UVB band, imaging of α contaminants is often performed under dark conditions, providing information about the location of the α contamination concerning the intensity of the radioactive source. The environment is then photographed under light conditions at the exact location and angle, providing coordinate information corresponding to the actual object. The two are imaged superimposed as the result of α imaging [50,92]. In 2001, Pineauu et al. [92] suggested that the α fluorescence effect could be used for imaging. However, due to the limitations of the imaging technology, researchers believed that directly using gases such as air as scintillation materials had significant limitations.

It was not until 2004 that Baschenko et al. [47] performed α-imaging experiments in dark conditions, where a ^239^Pu source of 37 MBq was photographed from 30 m away, and they studied the effect of γ particles on α particles and found that ^60^Co with an activity of 185 MBq placed next to ^239^Pu had essentially no effect on imaging.

With the significant development of imaging technology, in 2005, Lamadie et al. [4] used CCD imaging for in situ measurement of α particles, which could detect α particles at the MBq level at a distance of 1 m from the detector and 10 mm-thick Plexiglas locations apart, under dark conditions for 600 s

In 2013, Mahé et al. [93,94] investigated an α-imaging technique based on UVA/UVB bands for effective α-particle monitoring during decommissioning of nuclear facilities and nuclear accident emergencies, which can image radioactive sources in real-time, using ICCD with a dual microchannel plate enhancement unit, which improves the sensitivity to photons with a quantum of photons at wavelengths between 200 nm and 440 nm. The efficiency can be greater than 20%. It can work under the condition of light illumination of 10^−6^ lux. It can also provide real-time α warning for working gloveboxes and locate contaminants in gloveboxes with α radioactive contamination that need to be characterized and cleaned. Moreover, the data obtained from α and γ imaging can be combined to obtain a more accurate α contamination situation.

To investigate the effect of different imaging techniques on α imaging, in 2015, Sand et al. [58] first studied the performance of electron-multiplying CCD cameras (EMCCD) and enhanced CCD cameras (ICCD) in α imaging, using Andor products and comparison experiments also performed under dark conditions. The results show that the ICCD camera performs better than the EMCCD camera for detection in field conditions. At the same time, both cameras measured α radiation at 0.5 m from the detector for 100 s. Kerst et al. [19] conducted a study for a specific crime investigation scenario and achieved imaging of α contamination in dark conditions by designing an EMCCD-based imaging system that could determine the presence of α radioactive material at indoor crime scenes and determine its location.

**Figure 4:**
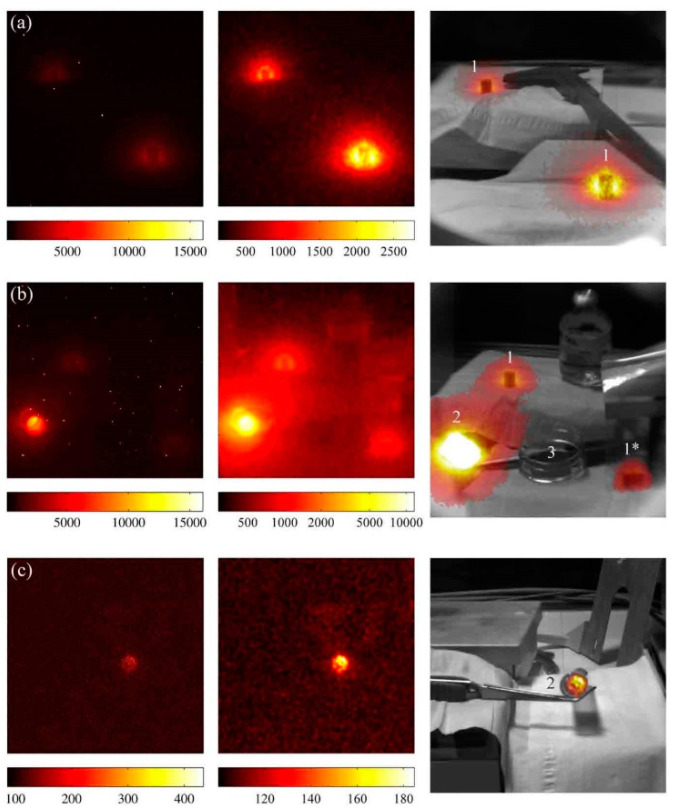
(**a**–**c**) The results of imaging α under three different conditions. The imaging is performed by superimposing the imaging of α in dark conditions on the imaging of the physical object in light conditions. The image on the left shows α imaging in the dark, while the image on the right shows the final image after superimposing the physical image in the light [58]. This figure is reprinted with the permission of Elsevier.

Meanwhile, Sand et al. pointed out that the new photocathode based on gallium nitride (GaN) can show good quantum efficiency below 360 nm, and its quantum efficiency decreases sharply for the rest of wavelengths [58]. With appropriate filtering modules, it can have high sensitivity to UV and good suppression and shielding effects on visible wavelengths, which is a very promising α-particle detector. Currently, a gallium nitride-based image intensifier developed by Hamamatsu in Japan is available [4]. An example of α imaging can be found in Figure 4.

From a simulated computational point of view, Thompson et al. [49] simulated the distribution of α particles ionized in the air using Geant4 based on their air fluorescence model AFM, where small and bright apertures are observed around the α source. The simultaneous presence of α-, β- and γ-emitting sources was also investigated for the case of simultaneous presence of α, β and γ sources, and the simulations yielded results that β sources form large and weak spots, and α sources are easier to localize compared to β sources. γ sources produce essentially uniformly distributed scintillation photons with low brightness at closer measurement distances, so the presence of α radioactive sources can be considered as background noise for filtering and removal.

### 5.2. α Imaging Based on the UVC Band

The DayCor SuperB UV camera developed by OFIL, which weighs about 3.5 kg with a minimum UV sensitivity of 3 × 10^−18^ w/cm^2^ and can detect a weak discharge of 1.5 pC at a distance of 8 m from the target, and enables the detection of moving targets [83,95], a performance that meets the requirements of UVC band-based day-blind α cameras [83]. The direct use of proven products without developing a separate UV camera for α imaging reduces the complexity of our work.

In 2011, Ivanov et al. [96] provided the results of actual α imaging using the DayCor SuperB camera. Moreover, they provided the detection limits of the instrument for α particles with energy of 5 MeV: the surface activity of the α particles that can be detected ranges from 40 Bq/cm^2^ to 100 Bq/cm^2^ when the distance between the detector and the α-emitting source is 3 m and the measurement time is 3600 s to 600 s. For a radioactive point source, the minimum detectable activity is 10^4^ Bq at a distance of 3 m. This work achieves daylight noncontact α imaging. Additionally, Ivanov et al. provide an outlook for future work: there is still a need for methodological studies on how to achieve searching and mapping of α contamination under more complex conditions of the source term, and there is a need to provide detection limits.

In 2021, Krasniqi et al. [97] proposed a method based on the UVC band that allows α detection and imaging in bright light, using a combination of ICCD imaging and photomultiplier tube scanning, along with enhanced luminescence using blown-in NO gas, which allows for the management and storage of α-containing radioactive source material at a safe distance.

The COROCAM 504 UV imager developed by COROCAM, also can achieve UV imaging in a sun-blind area. The camera can discharge at a distance of 10 m with a sensitivity of less than 0.75 pC, an overall weight of 2.2 kg, and an operating wavelength of 240−280 nm with a UV sensitivity of less than 2.05 × 10^−18^ w/cm^2^ and a built-in GPS module, which enables real-time imaging capabilities. The use of day-blind detectors and cameras based on the UVC band overcomes the interference from the background to a certain extent, but there are still some problems. First, the low number of photons in this band requires longer measurement times, higher activity of the α source, or closer detection distances to achieve better detection. Second, the band is not entirely free of background interference and still requires some filtering means to maintain the detection accuracy [91].

## 6. Conclusions

The detection of α particles is of great importance in nuclear energy and technology, and noncontact detection techniques based on the α-particle-induced UV fluorescence effect is a promising and practical method for α-particle detection, which has outstanding advantages over traditional methods.

The method is based on the scientific principle that α particles interact with matter to produce secondary electrons, which further excites air molecules, which are excited to produce spontaneous radiation. Since photons have more substantial penetration compared to α particles, they can be measured at an extensive range at a longer distance. Through the technical way of collecting and analyzing photon signals, information on the intensity and spatial location of α sources can be obtained, providing information and a basis for use in radioactive waste disposal, nuclear emergency response, nuclear facility decommissioning, nuclear explosion monitoring, military nuclear facility management, and nuclear security.

Research in recent years has focused on the following four areas: (1) α detection based on UVA/UVB bands; (2) α detection based on the UVC band; (3) α imaging based on UVA/UVB bands; (4) α imaging based on the UVC band.

Concerning the current technology and the complex reaction mechanisms of α particles in the air, it is challenging to achieve quantitative analysis and spatial localization at the same time. Therefore, two detection methods have been developed according to the focus, direct α detection, and α imaging techniques. The former mainly tends to detect and quantitatively analyze the α source without focusing on the location of the α source, and mainly uses photoelectric conversion equipment such as photomultiplier tubes or photodiodes to collect and process the UV signals, together with filtering means to achieve α detection. The latter is more concerned with the spatial information of the α source without focusing on quantitative analysis, and mainly uses imaging methods such as CCD, ICCD, or EMCCD, also through filtering and other processing methods, to realize the imaging of α particles.

As nitrogen is the main component of air, the current research on fluorescence effect is mainly focused on the fluorescence effect of nitrogen, and there are relatively few studies on the part of oxygen luminescence in air and its yield compared with the luminescence yield of nitrogen, and there is a lack of experiments on the broad-band spectral analysis of α-particle-induced fluorescence effect. At the same time, secondary electrons interacting with air molecules will also produce oscillating electrons, which are not used as monitoring objects because of the short-range of oscillating electrons, which is only about 10 nm.

UV light generated by nitrogen fluorescence in the air is mainly distributed in the UVA/UVB band. Since the background noise in this band is much larger than the characteristic UV signal in the actual detection environment, studies on the detection of UV light in the UVC band have also been carried out. Although the characteristic signal in this band is weak, it has essential research value because the noise in this band in the environment is similarly tiny. In order to reduce the difficulties in detection caused by characteristics such as high background noise interference and weak characteristic signals, both enhanced luminous efficiency and filtering to reduce background interference are usually used to improve the signal-to-noise ratio. Some research has been carried out in areas such as detection in dark environments or artificial light sources, adding gas purging to the detection environment, and conformal measurements.

All of the above four noncontact α-particle detection methods have advantages and disadvantages, and the appropriate detection technique can be selected according to the use scenario and application. For future work, it is believed that further research is needed in several areas, including, but not limited to, the following:

(1) Study of the effect of the content of water vapor in the air on the α-induced fluorescence effect.
(2) A study of ways to enhance luminescence efficiency. Comparison of the degree of enhancement of luminescence when different gases are used for the purge body.
(3) Optimization of the filtering method to reduce the interference of noise on the characteristic signal. The characteristic signal of α-particle radioluminescence can be distinguished from background signals such as the Sun.
(4) Optimization of methods for quantitative detection of α particles and study of analytical methods for uncertainty and detection limits.
(5) Optimization of nuclear electronics systems to improve the signal-to-noise ratio for direct detection and imaging.
(6) Research on UV-collecting materials and devices and their optimization, to improve the collection efficiency of UV photons.
(7) Study on the robustness and regularity of the luminescence yield with the change of air state. Quantitative measurement of α radiation in various complex environments is possible if the pattern of variation of luminescence yield with characteristics such as air temperature, humidity, or air pressure can be found.
(8) Nuclide identification using methods such as pattern recognition based on the nature of the source and the state of the air.
(9) Study of a method for accurate detection and imaging of complex types of pollution sources. For example, to study the extent to which the detection and imaging of α sources are affected by higher radioactivity β or γ sources.
(10) Study of the transmission of UV light by various translucent materials for use in correcting for the attenuation of UV light by translucent materials.
(11) Develop codes of practice and standards related to noncontact detection of α radiation.

## Figures and Tables

**Table 1:** Advantages and disadvantages of the main research methods.

		Strength	Weakness
Method	Detection technology	easy quantitative analysis; high sensitivity; cheap	not intuitive; cannot locate α contamination for a single detector
	Imaging technology	visualization of the α radiation field; easy to locate α contamination	inconvenient for quantitative analysis; poor sensitivity; expensive
Band of characteristic photons	UVA/UVB	high yield of photons; more sensitive in dark conditions	the background from the sun causes significant interference
	UVC	measurement under light conditions possible	low yield of photons
